# Institutional Laundries

**Published:** 1912-05-18

**Authors:** F. P. Carroll

**Affiliations:** Charing Cross Hospital, Agar Street, Strand, W.C.


### THE EDITOR'S LETTER-BOX.

### Institutional Laundries.

*To the Editor of* The Hospital.

Sir,—I was much interested in your article on "Institutional Laundries" in your issue of April 27, more especially with regard to what you say on the subject of water softening. The tremendous waste of soap due to hard water is a fact little realised by hospital officers. You give the Rudolf Virchow Hospital's experiments as showing that where one degree of hardness exists sixteen times the amount of soap is required to that necessary where the water is quite soft. Expressed in terms of pounds, this wastage (according to Mr. S. Rideal, D.Sc., a recognised authority) is one pound of soap per thousand gallons of water for every degree of hardness. The annual cost of this can be readily calculated by every hospital secretary according to the water consumption of his institution. And it must be remembered that ordinary London water has usually from 15 to 20 degrees of hardness.

The question of the laundry water, however, is exceeded in importance, I think, by that of the boiler feed water. The use of hard feed water results in increased expenditure in almost every direction. If the coating which forms on the boiler be allowed to remain, the waste of heat in raising steam is very great indeed. It is estimated that one-sixth of an inch of scale necessitates the use of 16 per cent. more fuel, a quarter of an inch 50 per cent., and half an inch 150 per cent. In other words, a hospital which burns normally 20 tons of coal a week will have to burn 30 tons to raise an equal amount of steam when a quarter of an inch of scale has accrued on the boiler. Taking coal at 16s. a ton, the consequent wastage through the use of hard water is £8 a week, and, of course, the evil is constantly being aggravated in intensity.

If, on the other hand, the scale be regularly chipped away, the cost of labour is not inconsiderable. A fortnight is a very moderate estimate of the time required by a man to chip the entire inner surface of a boiler. And it has to be remembered that chipping is very likely to injure the boiler and to start the rivets.

The question is complicated by the fact that a totally soft water is likely to corrode the fittings of a boiler. It is desirable, therefore, that feed water should not be softened below two or three degrees of hardness.

The cost of practically all the water-softening plants at present on the market is so high that no doubt hospital secretaries consider the capital outlay too large to be seriously considered. In point of fact, however, it may be doubted whether such an outlay is necessary, for I am led to believe, from experiments and appliances I have seen, that the cost of an adequate softener need not be more than about one-fifth of the price usually charged, while the cost of upkeep should be proportionately small. —I am, dear Sir, yours faithfully,

F. P. Carroll.

Charing Cross Hospital, Agar Street, Strand, W.C.

